# Genome-wide gene-based association study

**DOI:** 10.1186/1753-6561-3-s7-s135

**Published:** 2009-12-15

**Authors:** Hsin-Chou Yang, Yu-Jen Liang, Chia-Min Chung, Jia-Wei Chen, Wen-Harn Pan

**Affiliations:** 1 Institute of Statistical Science, Academia Sinica, Number 128, Section 2, Academia Road, Nankang, Taipei 115, Taiwan, Republic of China; 2 Institute of Biomedical Sciences, Academia Sinica, Number 128, Section 2, Academia Road, Nankang, Taipei 115, Taiwan, Republic of China

## Abstract

Genome-wide association studies, which analyzes hundreds of thousands of single-nucleotide polymorphisms to identify disease susceptibility genes, are challenging because the work involves intensive computation and complex modeling. We propose a two-stage genome-wide association scanning procedure, consisting of a single-locus association scan for the first stage and a gene-based association scan for the second stage. Marginal effects of single-nucleotide polymorphisms are examined by using the exact Armitage trend test or logistic regression, and gene effects are examined by using a *p*-value combination method. Compared with some existing single-locus and multilocus methods, the proposed method has the following merits: 1) convenient for definition of biologically meaningful regions, 2) powerful for detection of minor-effect genes, 3) helpful for alleviation of a multiple-testing problem, and 4) convenient for result interpretation. The method was applied to study Genetic Analysis Workshop 16 Problem 1 rheumatoid arthritis data, and strong association signals were found. The results show that the human major histocompatibility complex region is the most important genomic region associated with rheumatoid arthritis. Moreover, previously reported genes including *PTPN22*, *C5*, and *IL2RB *were confirmed; novel genes including *HLA-DRA*, *BTNL2*, *C6orf10*, *NOTCH4*, *TAP2*, and *TNXB *were identified by our analysis.

## Introduction

Genome-wide association study (GWAS) has been broadly applied to identify disease susceptibility genes of complex disorders. Single-locus association tests are routinely run to identify causal or associated single-nucleotide polymorphisms (SNPs) having strong marginal effects on disease status; however, their power to detect minor-effect SNPs may not be satisfactory. Multilocus association tests, which incorporate genetic information such as linkage disequilibrium (LD) and genetic distance, are performed to improve test power of single-locus association tests.

In order to analyze a large number of SNPs across the human genome, chromosomal regions on which to apply multilocus association tests should be defined in advance. Two frequently used procedures to define regions in a GWAS are the sliding-window approach and LD-block approach. A sliding-window approach defines regions by assigning a pre-determined window size or selecting a window size subject to an optimization criterion, and then a multilocus association test is performed in each window. It provides a convenient way to define regions and then scan each chromosome sequentially; however, the defined chromosomal segments may not have a biological function. An LD-block approach defines regions by determining LD/haplotype blocks, and then a multilocus association test is performed in each block. It uses a data-driven procedure to define blocks and then focuses on the examination of biologically meaningful blocks; however, use of different LD measures or block identification algorithms may obtain different blocks and hence draw different conclusions.

In this paper, we propose a two-stage genome-wide association scan, consisting of a single-locus association scan for the first stage and a gene-based association scan for the second stage. In comparison with a single-locus association test, the proposed method has the following merits: 1) biological information is incorporated into the definition of study regions, 2) tests are more powerful relative to single-locus association tests, 3) the multiple-testing problem is alleviated, and 4) the impact of genes can be evaluated directly and results are easier to interpret and generalize. Compared with a sliding-window approach, a gene-based approach contains richer information in a biological sense; compared with an LD-block approach, the regions analyzed by a gene-based approach are more stable and the analysis involves less intensive computation.

The proposed method was used to identify disease genes susceptible to rheumatoid arthritis (RA). We analyzed Genetic Analysis Workshop 16 Problem 1 RA data. The data consisted of 2,062 Illumina 550 k SNP chips from 868 RA patients and 1,194 normal controls collected by the North American Rheumatoid Arthritis Consortium [1]. Genotype data of 545,080 SNPs, which were probed on an Illumina 550 k SNP chip, were provided. A dichotomous disease status of RA and 530,720 autosomal SNP markers were analyzed in this GWAS.

## Methods

We illustrate the flow of the proposed two-stage genome-wide association scan as follows. At the first stage, we quantify trend effects of alleles for autosomal SNPs by calculating *p*-values of the exact version of the Armitage trend test [2], which is a powerful and valid association test even for analyses of rare-allele loci and Hardy-Weinberg-disequilibrium loci. The exact *p*-value of the *i*^th ^SNP is the sum of probabilities for the permutations with statistics at least as extreme as the observed statistic. A logistic regression can be carried out if genetic and/or environmental effects should be adjusted.

At the second stage, we carry out a genome-wide gene-based association scan. All SNPs are divided into two types: inter-gene SNPs and intra-gene SNPs according to the annotation information. Inter-gene SNPs are treated as singletons, and their *p*-values and the corresponding physical positions are denoted as  and , respectively. Intra-gene SNPs within the same gene are bound as an SNP cluster, and the *p*-value of the *s*^th ^intra-gene SNP within the *t*^th ^gene and the corresponding physical position are denoted as  and , respectively. We use physical position of the first SNP within a gene to represent the gene location for a result display in the Results section.

To evaluate total effects of genes (SNP clusters) on RA, we combine *p*-values of intra-gene SNPs within a gene by using the truncated product *p*-value method [3]. The combination is based on multiplication of *p*-values, less than some pre-specified cut-off threshold, from single-locus association tests. The test statistic for the *t*^th ^gene is defined as:

where *θ *is a threshold of p-value truncation. The cumulative distribution function of *Z*_*t *_is:

*p*-Values of genes and the corresponding physical positions are denoted as  and . In accordance with the sorted physical positions,

*p*-values of inter-gene SNPs and genes are arranged in order. Finally, false-discovery rate (FDR) correction [4] is applied to all *R*+*T p*-values to adjust for multiple testing.

## Results

We calculated exact *p*-values of the Armitage trend test for 530,720 autosomal SNPs in the study of RA. According to the Illumina 550 k SNP-chip annotation file, all SNPs were partitioned into 285,823 inter-gene SNPs and 244,897 intra-gene SNPs, which were located on 15,635 genes. The truncated product *p*-value statistics with *θ *= 0.05 in Eq. (1) and the empirical *p*-values were calculated for 15,635 genes.

**Figure 1:**
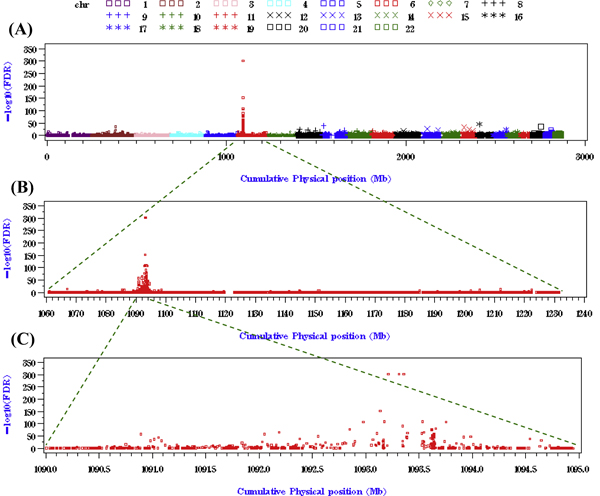
**Genome-wide gene-based association mapping of RA data**. The horizontal axis denotes the physical position of SNPs (scale in Mb) and the vertical axis denotes the FDR-adjusted *p*-values (scale in -log_10_). A, FDR-adjusted *p*-values (scale in -log_10_) of genome-wide gene-set association tests; B, Association signals on chromosome 6; C, Association signals on a chromosomal region between 1,090 Mb and 1,095 Mb (in cumulative physical position).

We removed 1,088 SNPs with a minor allele frequency of zero, resulting in the removal of 1,078 inter-gene SNPs and 10 genes. In total, 300,370 *p*-values of genes and inter-gene SNPs were sorted according to their physical positions. FDR was applied to these *p*-values and FDR-adjusted *p*-values in -log_10 _scale, -log_10_(*P*_FDR_), were displayed (see Figure 1(A)). A high peak of association signals was observed on chromosome 6 (symbol: red square). Zooming in to chromosome 6, we found a 5-Mb region with strong association signals (see Figure 1(B)). Further zooming in to the 5-Mb region (1,090 Mb-1,095 Mb in cumulative physical position), 55 out of 57 genes/SNPs across the human genome satisfying -log_10_(*P*_FDR_) > 40 were located in this region (see Figure 1(C)). In addition, the top three association signals were found at *C6orf10*, *BTNL2*, and *HLA-DRA*. Their individual -log_10_(*P*_FDR_) values were as high as 302.65.

The -log_10_(*P*_FDR_) values of the top 100 significant loci were all greater than 20 (data not shown). Among the 100 loci, 86 genes/SNPs were located in a region between 30,041,240 bp and 33,797,498 bp on chromosome 6. The region overlapped with the human major histocompatibility complex (MHC) region, which is well known proven to be one of the most important genomic regions related to RA [5]. There were 14 genes on other chromosomes, and some have already been proven to be associated with RA. For example, gene *C5 *had a -log_10_(*P*_FDR_) of 20.46 in our study, and this gene has been shown to be an RA-associated gene [1].

We compared the results of single-locus association tests and gene-based association tests under two thresholds of significance, -log_10_(*P*_FDR_) = 3 and -log_10_(*P*_FDR_) = 7. For a threshold of -log_10_(*P*_FDR_) = 3, the exact Armitage trend tests identified 433 SNPs among a total of 529,632 SNPs and gene-based association tests identified 849 genes/SNPs among a total of 300,370 genes/SNPs. In total, 463 genes/SNPs were identified by gene-based association tests but failed to be detected by single-locus association tests. On the other hand, 69 intra-gene SNPs revealed by the exact Armitage trend tests failed to be identified by gene-based association tests, including 65 SNPs located on individual genes and 2 SNP pairs located on two individual genes. For a threshold of -log_10_(*P*_FDR_) = 7, the exact Armitage trend tests identified 141 SNPs among a total of 529,632 SNPs and gene-based association tests identified 308 genes/SNPs among a total of 300,370 genes/SNPs. We found that 157 genes/SNPs found by gene-based association tests failed to be identified by the exact Armitage trend tests; however, only 10 intra-gene SNPs identified by the exact Armitage trend tests but not by gene-based association tests, where all of the 10 SNPs were on individual genes. The intra-gene SNPs missed by gene-based association tests were not in the list of top 100 genes/SNPs.

We compared our results with other studies. Association of two previously reported genes [1,6], *PTPN22 *and *IL2RB*, were confirmed by our method. The adjusted *p*-value -log_10_(*P*_FDR_) of the two important genes were 13.29 and 4.75 in our study, respectively. We also compared our results with the other contributions in GAW16 Group 16 - Gene- or region-based association tests. In spite of the use of various methods and procedures, some consistent results were obtained. The genes included *AGPAT1 *(62.04) [7], *HLA-C *(65.47) [8], and *PHF19 *(10.21) [9], where the numbers in parentheses were -log_10_(*P*_FDR_) in our study. In addition, we also identified some novel RA-associated genes/SNPs that have not been reported before, for example, *NOTCH4*, *TAP2*, and *TNXB*. The adjusted *p*-value -log_10_(*P*_FDR_) of the three genes were 153.79, 108.33, and 108.09 in our study, respectively. The roles of these genes/SNPs in RA are not clear and merit further study.

To consider strong effects of HLA genes and extensive LD in the human MHC region, we replaced the exact Armitage trend test at the first stage with a logistic regression model adjusting for the status of shared-epitope alleles. After adjusting for the effect of *DRB1 *shared-epitope alleles, we found that the results of the top three loci, *C6orf10*, *BTNL2*, and *HLA-DRA*, remained the same, and all of the aforementioned genes were still highly significant. The major difference was that 44% of the top inter-gene SNPs in the human MHC region were not longer significant after the adjustment of shared-epitope alleles.

## Discussion

Under the proposed two-stage association mapping framework, there are different methods that can be applied to integrate SNP information within a gene, for example, combination of test statistics, principal-components analysis, and multiple regression analysis. This paper considers a *p*-value combination, which has been broadly used in a GWAS [3,10,11]. SNPs in a disease-gene region are more likely to present association signals compared with SNPs in a disease-gene-free region. Therefore, combination of the *p*-values will strengthen association signals and increase power of association tests in a disease-gene region. However, this method may miss a relatively small number of intra-gene SNPs that can be detected by single-locus association tests. The proposed gene-based association test provides a powerful alternative but is not intended to substitute for a single-locus association test.

Unlike some researchers who have performed *p*-value combination in sliding windows [3,10,11], we combine *p*-values to evaluate a total impact of SNPs within each gene in a GWAS. There are multiple types of *p*-value combination methods. This paper considers a truncated product *p*-value statistic because of its good performance in our previous simulation study [12,13]. However, in the analysis of RA data, we also calculated empirical *p*-values of different combination methods including the minimum *p*-value statistic and Fisher's product *p*-value statistic for the top significant genes. All of the methods obtained similar empirical *p*-values, implicating the strong association of the identified genes.

An extended application of *p*-value combination methods is to study biological pathways or protein networks of complex diseases. *p*-Values of SNPs within genes involved in a pathway/network can be combined to evaluate the global effect of a pathway/network and then used to identify disease-specific pathways and networks. The applications highlight the potential of *p*-value combination methods in genetic/genomic dissection of complex diseases.

Strong effects of HLA genes on RA and extensive LD in the human MHC region, where the genes are located, are issues that should be taken into consideration in the analysis of the RA data. An analysis that does not consider the issues may overstate genetic association in this region. To circumvent the issues, an alternative approach may be to replace the exact Armitage trend test at the first stage with a logistic regression model containing covariates of the HLA loci and/or SNPs in LD with the HLA loci. Marginal effects of tested genes/SNPs can be evaluated independently after conditioning out the effects of LD and HLA genes. We only adjust the status of *DRB1 *shared-epitope alleles in this paper and the analysis can be further enhanced by considering additional information on LD structure and HLA genes in the future.

## Conclusion

This study introduces a two-stage genome-wide gene-based association scanning procedure. Compared with some existing single-locus and multilocus methods, this method has practical merits in aspects of biology, computation, and statistics. We applied this method to analyze GAW16 Problem 1 RA data. Compared with the results from other RA association studies, our analysis not only successfully confirmed association of previously reported genes but also identified novel RA-associated genes/SNPs.

## List of abbreviations used

FDR: False-discovery rate; GWAS: Genome-wide association study; LD: Linkage disequilibrium; MHC: Major histocompatibility complex; RA: Rheumatoid arthritis; SNP: Single-nucleotide polymorphism.

## Competing interests

The authors declare that they have no competing interests.

## Authors' contributions

H-CY conceived the experimental design and statistical methods and prepared the manuscript. Y-JL performed data analysis. C-MC prepared gene annotation. J-WC and W-HP contributed to the discussion and preparation of the final manuscript with H-CY. All authors have approved the final manuscript.
